# USING CANCER SURVIVORSHIP EXPERIENCES TO INFORM CANCER ADVOCACY IN NIGERIA

**DOI:** 10.1093/geroni/igae098.3249

**Published:** 2024-12-31

**Authors:** Chizobam Nweke, Chinelo Nduka, Runcie Chidebe, Candi Nwakasi

**Affiliations:** University of Connecticut, Storrs, Connecticut, United States; Nnamdi Azikiwe University Teaching Hospital, Nnewi, Anambra, Nigeria; Miami University, Oxford, Ohio, United States; University of Connecticut, Storrs, Connecticut, United States

## Abstract

**Background:**

Despite having a high cancer burden that is still rising, Nigeria, like many rapidly aging countries in sub-Saharan Africa, due to a very high burden of infectious diseases, is not paying adequate attention to non-communicable diseases, especially cancer. This study explored the experiences of cancer survivors for informing tailored cancer advocacy programs in Nigeria.

**Methods:**

A qualitative descriptive approach was used on a sample of 19 women with age ranging from 29 – 55 years, with a history of breast cancer. We had four focus group discussions during data collection and used reflexive thematic analysis for data analysis.

**Results:**

The following themes were identified from the analysis: 1) strengthening care quality, 2) addressing delayed presentation, 3) expanding access to informal support, and 4) alleviating the cost burden of cancer care.

**Discussion:**

A better understanding of cancer survivorship experiences among Nigerian women can help strengthen cancer advocacy in Nigeria. In turn, strong cancer advocacy will help to prioritize cancer control, and mitigate a looming cancer crisis in Nigeria.

